# Fever: Its Pathology and Treatment by Antipyretics

**Published:** 1891-06

**Authors:** 


*Fever: its Pathology and Treatment by Antipyretics.* By HOBART AMORY HARE, M.D., B.Sc. Pp. 166. Philadelphia: F. A. DAVIS. 1891.

This book, which gained the Boylston Prize at Harvard University in 1890, gives a useful account of the modern antipyretics—antipyrin, antifebrin, thallin, phenacetin, and salicylic acid—and of the physiological and clinical observations upon which their administration is based. We think the author would have done well to have retained the original title of the essay; namely, "The Uses and Values of Antipyretics," as the subject of fever is dismissed very briefly, and no serious attempt is made to discuss its pathology in an adequate manner. Dr. Hare has himself conducted many calorimetric experiments with these drugs. Some of his results have been adversely criticised by Prof. Wood, and he here defends them. He states clearly and impartially the researches, clinical and experimental, of different authors; so that the reader is enabled to estimate the value of the evidence upon which the conclusions arrived at are based. While rightly pointing out that in using antipyretics we treat a symptom and not the specific process, he hardly brings out sufficiently clearly the definite object which is held in view in the direct treatment of excessive or long-continued pyrexia; namely, to prevent the deleterious action of such pyrexia on muscular and glandular tissue. Antipyrin is given the first place, both as an antipyretic and an analgesic; personally we should give antifebrin or phenacetin the preference as antipyretics, whilst allowing that antipyrin is the most efficient analgesic. The author thinks that thallin has been undeservedly neglected; but the reason surely is, that it has been found to be neither so safe nor so efficient as the others. The book contains much information in a convenient form for reference; and now that these drugs have come into general use, such an account of their pharmacology and therapeutic application as is here given will doubtless be found of service.

